# Perception of primary care physicians on the impact of comprehensive geriatric assessment: what is the next step?

**DOI:** 10.1186/s13584-016-0106-3

**Published:** 2016-10-01

**Authors:** Pei Chen, Michael A. Steinman

**Affiliations:** 1 Division of Geriatrics, Department of Medicine, University of California, San Francisco, 3333 California St, Suite 380, San Francisco, CA 94143 USA; 2 San Francisco VA Medical Center, 4150 Clement St, San Francisco, CA 94121 USA

**Keywords:** Comprehensive geriatric assessment, Primary care, Consultative medicine, Medical education

## Abstract

Older adults are at high risk of developing multimorbidity, and the high levels of clinical and psychosocial complexity in this population pose special challenges for primary care physicians (PCPs). As a way to improve the care for the older adults, a number of health systems have developed programs to provide comprehensive geriatric assessment (CGA), which generally refers to an intensive interprofessional evaluation and management of geriatric syndromes with the goals of maximizing health in aging. Sternberg and Bentur examined the impact of CGA as perceived by PCPs, the PCPs attitude toward CGA, and their satisfaction with CGA. In this commentary, we seek to provide additional context to the current state of outpatient consultative CGA and how it relates to the findings in the study by Sternberg and Bentur. The knowledge gained from this study begs for future investigations, especially in the areas of PCPs’ understanding of outpatient consultative CGA, the perceived benefit in health outcomes and actual health outcomes, perceived needs in geriatric consultation, preference in management of complex geriatric syndromes, and interests in continuing education in geriatrics. Insight into these factors could allow for improvement of the current outpatient consultative CGA model and allow for adaption of the model to local needs.

## Background

Older adults are at high risk of developing multimorbidity, and the high levels of clinical and psychosocial complexity in this population pose special challenges for primary care physicians (PCPs). As a way to improve the care of older adults, a number of health systems have developed programs to provide comprehensive geriatric assessment (CGA), which generally refers to an intensive interprofessional evaluation and management of geriatric syndromes with the goal of maximizing health in aging. This approach is intuitive, but gaining understanding of how outpatient consultative CGA is perceived by relevant stakeholders and how it affects meaningful health outcomes could influence implementation and dissemination of the model.

In this context, Sternberg and Bentur sought to examine rarely studied elements of CGA- how PCPs who referred patients to outpatient consultative CGA perceived its impact and contribution to their care of the older adults, and these PCPs’ attitudes toward and satisfaction with outpatient consultative CGA [1]. They surveyed PCPs in Israel who had referred at least six patients for CGA in the year prior. The majority of the referrals to CGA were for cognitive impairment and rapid functional decline. More than half of the participating PCPs thought that CGA was “very useful” for diagnosis and treatment of cognitive decline and dementia and for confirming diagnoses. In general, PCPs had positive attitudes toward CGA compared to their attitude toward other consultative clinics, with half or more of the PCPs “definitely agreeing” that CGA was better than other consultative clinics at approaching patients holistically, providing patient-centered medication recommendations, and offering detailed guidance. However, PCPs, especially those with an internal medicine background, were less satisfied with CGA recommendations. Only about one-third of the PCPs stated they were “very satisfied" with the guidance CGA clinics provided to PCPs, patients, and families, including recommendations for medications, treatments and social assistance services, and guidance to patients and families on how to live safely at home and utilize social services. Furthermore, only 15 % of the PCPs thought that their patients are more willing to comply with the CGA recommendations than recommendations received from other specialty clinics.

To understand the meaning of the results found in the Sternberg and Bentur study, it is important to examine the evolution and context of outpatient consultative CGA.

## Comprehensive geriatric assessment: history, challenges, and opportunities

The concept of CGA first appeared in the professional literature in the 1980s, and the impact of CGA was first evaluated in the setting of an outpatient consultative clinic in a health maintenance organization (HMO) which showed increased identification of geriatric syndromes and advice for changes in medication regimens [2, 3]. This early work showed short-term benefits in cognitive function, but no long term benefits in cognitive function and other health outcomes, which may be the result of limited follow up by CGA programs [3].

After initial studies in the ambulatory HMO setting, CGA was adapted into other health care settings, including inpatient geriatric and rehabilitation units, inpatient consultation, and post-discharge home visits. A randomized controlled trial and a meta-analysis looked into adaptation and outcomes of CGA and revealed programs that had control over medical recommendations and extended ambulatory follow up tended to be more effective, and that impact on mortality was more robust in inpatient than outpatient settings [4, 5]. Costs of outpatient consultative CGA were similar to other common medical interventions, which could encourage the dissemination of the model [6]. Consultative CGA for PCPs has since evolved and translated into other subspecialties, such as oncology, as a way to assess prognosis and tailor treatment plans for frail older adults [7, 8].

As noted above, limited control over implementing care recommendations has handicapped the potential effectiveness of CGA. What then might be done to improve follow-through of recommendations to maximize potential benefit? One logical approach is closer follow-up and longitudinal engagement with patients and referring clinicians. For example, Reuben et al. demonstrated an outpatient consultative CGA program that used telephone and written communications with the PCPs, reviewed recommendations with the patients, and used telephone follow-up with patients had meaningful benefits in forestalling declines in functional status and health-related quality of life [9].

Another key step for improving CGA is to learn about the attitudes and experiences of stakeholders who use CGA, namely patients, caregivers, and referring clinicians who seek guidance from CGA programs about how to better care for their patients. Understanding these perceptions is critical for at least two reasons. They are important mediators through which the CGA recommendations are translated into concrete actions. They also provide valuable insights into the strengths and failings of outpatient consultative CGA programs and how they might be improved.

There is a limited body of research on perceptions and attitudes of patients and caregivers toward CGA, which move us incrementally toward the goal of understanding the stakeholders’ perceptions. Older adults have expressed appreciation toward CGA because of how it helped to increased their knowledge of their own conditions and reduce stress as related to their illness. However, they also expressed a wide range of emotions, including anxiety and the feeling of threat, related to the CGA process and the impact of CGA on their lifestyles and living arrangements [10]. In comparison with older adults, family and caregivers typically had a more positive view of CGA, reporting not only increased knowledge and reduced stress as a result of CGA, but also enhanced skills, improved perceived competence, better communication, improved decision-making, greater access to services, and positive health outcomes [10, 11]. The families’ and caregivers’ perception of CGA outcomes met their expectation of CGA and their goal of understanding the patients’ evaluation and care plans through CGA [11]. The positive experience of the family and caregivers is also reflected by the finding from Sternberg and Bentur’s study in which the PCPs also felt that the support and counseling from CGA enhanced their abilities to counsel patients and families on getting help and services [1].

Because PCPs are also key stakeholders who refer patients to outpatient consultative CGA and implement CGA recommendations, understanding their perceptions is critical as well. In this light, several findings by Sternberg and Bentur are noteworthy, particularly the apparent gap between relatively high rates of agreement that outpatient consultative CGA was holistic, patient centered, and provided detailed recommendations, and the relatively lower rates of satisfaction about recommendations for management and the guidance provided to older adults and their family members [1]. It is important not to over-interpret the gap– it is substantial but not profound, and these survey items are not directly comparable, since Sternberg and Bentur assessed attitudes with a reference-based agreement scale and satisfaction with a freestanding satisfaction scale. Yet, this observation does raise the question of whether recommendations are perceived as less useful because they are overly complex, difficult to implement, or not immediately actionable. Communication was also reported as being far from ideal: only 20-24 % of referring PCPs “definitely agreed” that the CGA clinic is more considerate of the patients’ wishes and better at communication with PCPs than other consultative clinics, and only 36 % of the PCPs were “very satisfied” with communication with the CGA physicians and staff [1]. Since the study was only conducted among PCPs who had made at least six referrals in the prior year, these relatively low rates of agreement and satisfaction might have been even lower if physicians who did not refer their patients to CGA had also been included. In this respect, the relatively poor ratings on how considerate CGA clinics were of patients’ wishes, as well as suboptimal marks for communication, are likely holding back the effectiveness of the CGA clinics studied by Sternberg and Bentur.

These challenges with communication, usefulness of recommendations, and what is known from prior research about the importance of follow-up are likely closely interrelated. Sternberg and Bentur reported that only 15 % of clinicians felt that their patients were more willing to comply with CGA recommendations than recommendations from other clinics [1]. In one of the very few other studies on this topic, Maly et al. found that the patients’ requests to follow CGA recommendations, the perceived patients’ wish, and the perceived cost-effectiveness of the CGA recommendations promoted the PCPs’ adherence to implement the recommendations [12]. More ongoing communication and follow-through with PCPs, to help them implement and troubleshoot recommendations, may also help close the satisfaction gap. As shown in another study, limited time and reimbursement contribute to reluctance to refer patients to consultative CGA, knowing that the recommendations will require more time commitment downstream or lead to non-adherence to the CGA recommendations as a way to self-preserve and prevent burnout of the PCPs [13]. However, the same study demonstrated that PCPs who had some geriatric training or exposure reflected positively on how the training changed their ways of caring for older adults, and they tend to embrace the more holistic approach and collaborative team effort [13].

## Conclusions and future directions

Maximizing the effectiveness of CGA will require a multipronged approach that involves closer engagement and follow-through with patients and PCPs and provides geriatric education to PCPs so that they are more receptive and better equipped to implement CGA recommendations. Several elements may facilitate this goal. First, fulfilling the perceived needs of patients, their families, and caregivers and empowering them to become their own advocates will not only improve uptake by patients but will also promote PCPs’ acceptance and adherence to CGA recommendations. Second, the development of an adherence program that not only follows up with the PCPs and patients after the initial consultation but also help PCPs understand the potential clinical value and cost-effectiveness of outpatient CGA may also increase adherence, since both have been shown to promote PCPs’ adherence. Lastly, improving continuing geriatric education for PCPs, both through traditional models and case-based learning from specific consultations, would improve PCPs ability to directly care for their older patients, facilitate adherence and implementation of CGA recommendations, and promote collaborative teamwork which is the foundation of geriatric care [13–15]. These changes will be neither quick nor easy to implement. But, if planned and done properly, they could create a virtuous cycle of improvement, whereby engaged patients will prompt their PCPs to implement CGA recommendations, and engaged and “geriatricized” PCPs will better counsel and help their vulnerable older patients to implement CGA recommendations.

## Abbreviations

CGA: Comprehensive geriatric assessment
HMO: Health maintenance organization
PCPs: Primary care physicians
